# Different Reactive Oxygen Species Lead to Distinct Changes of Cellular Metal Ions in the Eukaryotic Model Organism *Saccharomyces cerevisiae*

**DOI:** 10.3390/ijms12118119

**Published:** 2011-11-18

**Authors:** Ming J. Wu, Patrick J. O’Doherty, Patricia A. Murphy, Victoria Lyons, Melinda Christophersen, Peter J. Rogers, Trevor D. Bailey, Vincent J. Higgins

**Affiliations:** 1 School of Biomedical and Health Sciences, College of Health and Science, University of Western Sydney, Locked Bag 1797, Penrith South DC, New South Wales 1797, Australia; E-Mails: p.odoherty@uws.edu.au (P.J.O.); 16175596@student.uws.edu.au (P.A.M.); v.lyons@uws.edu.au (V.L.); t.bailey@uws.edu.au (T.D.B.); 2 Carlton and United Breweries, Fosters Group, 4-6 Southampton Crescent, Abbotsford, Melbourne 3067, Australia; E-Mails: melinda.christophersen@fostersgroup.com (M.C.); peterjohnrogers@me.com (P.J.R.); 3 School of Science, Griffith University, Nathan, Queensland 4111, Australia; 4 Ramaciotti Centre for Gene Function Analysis, School of Biotechnology and Biomolecular Sciences, University of New South Wales, NSW 2052, Australia

**Keywords:** reactive oxygen species, metal ions, ionomic profiling, yeast, *Saccharomyces cerevisiae*

## Abstract

Elemental uptake and export of the cell are tightly regulated thereby maintaining the ionomic homeostasis. This equilibrium can be disrupted upon exposure to exogenous reactive oxygen species (ROS), leading to reduction or elevation of the intracellular metal ions. In this study, the ionomic composition in the eukaryotic model organism *Saccharomyces cerevisiae* was profiled using the inductively-coupled plasma optical emission spectrometer (ICP-OES) following the treatment with individual ROS, including hydrogen peroxide, cumen hydroperoxide, linoleic acid hydroperoxide (LAH), the superoxide-generating agent menadione, the thiol-oxidising agent diamide [diazine-dicarboxylic acid-bis(dimethylamide)], dimedone and peroxynitrite. The findings demonstrated that different ROS resulted in distinct changes in cellular metal ions. Aluminium (Al^3+^) level rose up to 50-fold after the diamide treatment. Cellular potassium (K^+^) in LAH-treated cells was 26-fold less compared to the non-treated controls. The diamide-induced Al^3+^ accumulation was further validated by the enhanced Al^3+^ uptake along the time course and diamide doses. Pre-incubation of yeast with individual elements including iron, copper, manganese and magnesium failed to block diamide-induced Al^3+^ uptake, suggesting Al^3+^-specific transporters could be involved in Al^3+^ uptake. Furthermore, LAH-induced potassium depletion was validated by a rescue experiment in which addition of potassium increased yeast growth in LAH-containing media by 26% compared to LAH alone. Taken together, the data, for the first time, demonstrated the linkage between ionomic profiles and individual oxidative conditions.

## 1. Introduction

Oxidative stress, induced by either endogenous or exogenous oxidants, is the most common stress condition encountered by aerobic organisms like yeast and human beings. Endogenous reactive oxygen species (ROS) such as the hydroxyl radical (OH^•^), superoxide anion (O_2_^•^), hydrogen peroxide (H_2_O_2_), lipid peroxide and peroxynitrite frequently result from cellular metabolism through the incomplete reduction of oxygen in the mitochondria. Exposure to exogenous ROS such as the sulfhydryl-oxidising agent diamide also imposes a serious challenge to an organism’s survival. It is the reduction capacity of cellular antioxidant defence systems such as the thiol-containing glutathione that is critical to the elimination of these threatening oxidants [1]. However, the antioxidant balance can be disrupted at a particular temporal and spatial point if the amount of ROS overwhelms intracellular antioxidants. When this happens, the cellular survival response kicks in at the genomic level leading to changes in metabolism, that is, changes in metabolic pathways, nutrient levels and so on. As a result, the cells adapt and survive; or else they die.

Metal ions are the major components of the ionome which is defined as the overall cellular ion composition of a tissue or an organism. They have important roles in the cell’s survival or apoptotic process under physiological and stressful conditions. Essential ions such as Na^+^, K^+^, Mg^2+^, Ca^2+^, Zn^2+^, Fe^2+/3+^, Cu^+/2+^ and Mn*^n^*^+^ are indispensable to cell growth, whilst non-essential or toxic metal ions such as cadmium (Cd^2+^) are detrimental. In living organisms, ions are imported and exported in response to cellular metabolism. Ionomic homeostasis or dynamic equilibrium is tightly maintained in the cell by reacting to ion deficiency as well as ion overload. Essential ions are involved in almost every cellular function such as biosynthesis, energy production and proliferation by functioning as enzyme cofactors and messengers in signal transduction cascades [2]. Maintenance of the ionomic homeostasis is essential for cellular health. Any pronounced imbalance could lead to cell dysfunction. For example, zinc deficiency impairs yeast growth while excess zinc becomes toxic [3], and over-exposure to aluminium and copper is detrimental to brain function [4].

In concert with ROS, transition metal ions such as Fe^2+/3+^ and Cu^+/2+^ can trigger oxidative stress [5]. However, there is lack of evidence on whether oxidative stress condition can result in changes of elemental ion levels. The aim of this study is to delineate effects of oxidants on elemental ion composition using the model organism *Saccharomyces cerevisiae*. On the basis of the findings obtained by the means of transcriptomics and deletion mutant screening that cells have distinct molecular mechanisms to maintain protection against different ROS [6,7], we hypothesise that different oxidants may cause varying changes in ionomic profiles.

The advent of ICP spectroscopy has made ionomic measurement high throughput and cost-effective compared with transcriptomics and proteomics. The ionomic composition of common elements (Zn^2+^, Fe^2+/3+^, Cu^+/2+^, Mn*^n^*^+^, Cd^2+^, K^+^, Na^+^, Mg^2+^ and Ca^2+^) in yeast has been investigated under normal physiological conditions using deletion mutants [8,9]. However, Al^3+^ has not been studied in detail so far, in spite of its abundance and potential health hazard. In this study, we investigated ionomic profiles by the means of ICP-OES for Zn^2+^, Cu^+/2+^, Na^+^, Fe^2+/3+^, Mn*^n^*^+^, Mg^2+^, Al^3+^, K^+^ and Ca^2+^ in yeast cells treated with seven oxidants, including three peroxides [hydrogen peroxide, cumen hydroperoxide (CHP), linoleic acid hydroperoxide (LAH)], and the superoxide-generating agent menadione, the thiol-oxidising diamide [diazine-dicarboxylic acid-bis(dimethylamide)], thiol sulfenic group modifying agent—dimedone (5,5-dimethyl-1,3-cyclohexanedione), and a reactive nitrogen species—peroxynitrite. Subsequent analysis identified several elements which were elevated or reduced under specific oxidative conditions.

## 2. Results and Discussion

### 2.1. Determination of Arresting Concentrations of the Oxidants

**Figure 1:**
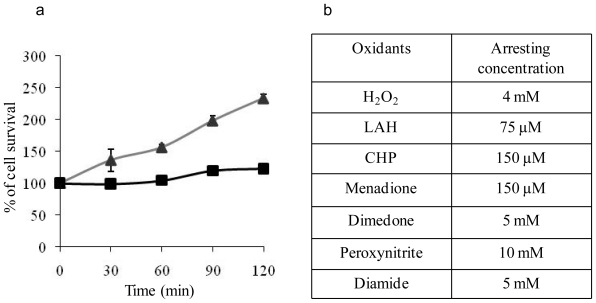
Arresting concentrations of oxidants for *S. cerevisiae* BY4743. (**a**) A representative example of yeast growth arrest under LAH-induced oxidative stress. The growth of BY4743 yeast cells treated with 75 μM LAH (■) and without the oxidant (▴) were measured by the percentage of cell survival at each of the time points relative to the starting total cell number at the zero time point. The data represent the means of six replicates for each time point with standard deviation shown; (**b**) Arresting concentrations for all seven oxidants determined in the same way as LAH were shown.

The elemental composition of an organism is a dynamic network relevant to the environmental changes [10]. The variation in ionomic profiles under a range of experimental conditions could reflect the physical integrity of the cells and possibly the status of cellular metabolism. The ionomic profiling of this study, carried out under arresting concentrations of oxidants in *Saccharomyces cerevisiae*, provided insights into the effects of individual oxidants on eukaryotic cells. The chosen biological condition, namely the temporary growth arrest, is designed to maximise the chance to detect any ionomic changes incurred by a given oxidant, since near normal cell growth under a low dose or excessive toxicity with a higher dose of the oxidant would either mask any variations upon treatment or undermine the physiological relevance of experimental findings. The arresting concentrations of the seven oxidants were determined as shown in Figure 1. A representative arresting curve was provided for 75 μM LAH (Figure 1a). Following 75 μM treatment, the yeast growth was arrested while the growth of non-treated yeast had doubled by 90 min. The arresting concentrations for the other six oxidants are listed in the table (Figure 1b). Such a phenotypic growth arrest of yeast upon oxidative exposure is apparently due to cell cycle progression delay [11–14]. During this delay, cells mount antioxidant defences to counter oxidant damage before resumption of the cell cycle and cell proliferation. Excessive oxidant, *i.e.*, a greater concentration than the arresting concentration, causes cell death, making any experimental observation almost irrelevant in a physiological context. In this study, the arresting concentrations for the six oxidants were determined at the cell concentration of OD_600_ 1.0 when the cells were treated. An OD_600_ 1.0 provides cells locked into the logarithmic phase of growth and also, importantly adequate cell mass for the subsequent ionomic profiling step.

### 2.2. Ionomic Profiling Under Seven Individual Oxidants

**Figure 2:**
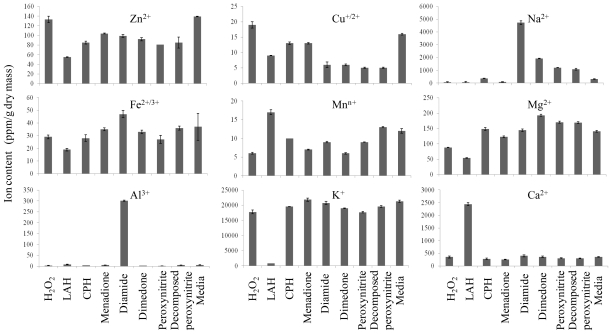
Element content in yeast samples under individual oxidative conditions. Yeast samples from each treatment (H_2_O_2_, LAH, CHP, menadione, diamide, dimedone, peroxynitrite, decomposed peroxynitrite and media control) were washed and then followed by ICP-OES measurement for nine metal ions as shown. The element content was expressed as ppm/g dry mass. The decomposed peroxynitrite was used as a specific control for peroxynitrite treatment because of its alkaline pH. Error bars indicate standard deviation of two biological replicate measurements.

In parallel to the diverse and unique molecular responses to varying oxidative conditions at the gene expression level [6,7,15], pronounced variations against the particular oxidants at the element level were observed in this study (Figure 2). Aluminium content after diamide treatment was 50-fold higher than the control, while Na^+^ content was 6-fold higher. Ca^2+^ changed little irrespective of ROS types apart from LAH which resulted in a nearly 7-fold increase of the intracellular Ca^2+^. An explanation of this finding is that LAH induced oxidative stress and membrane disruption, which might in turn lead to Ca^2+^ influx, since a strong rise of intracellular Ca^2+^ concentration was found in endothelial cells upon LAH exposure [16], which stimulated phospholipase activities, triggering phospholipid hydrolysis. The potential damage to the plasma membrane by LAH-induced Ca^2+^ influx might be linked to the next finding, namely that potassium ion (K^+^) was markedly depleted in LAH-treated yeast cells by a 26-fold reduction in comparison with solvent-treated control cells. This strongly indicated that the cell membrane was perturbed by LAH which led to the loss of intracellular K^+^, because the homeostasis of intracellular K^+^ is maintained by plasma membrane permeability and the cation-related membrane transporters [17]. Interestingly, peroxynitrite did not incur significant changes in ions tested in comparison to the decomposed peroxynitrite control (Figure 2).

**Figure 3:**
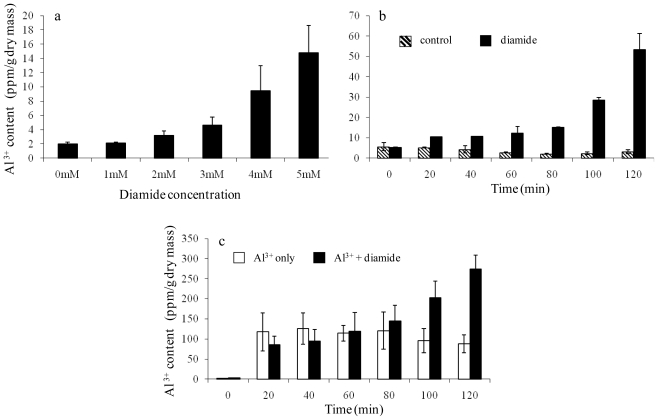
Characterization of Al^3+^ accumulation under diamide treatment. (**a**) Al^3+^ accumulation over a range of diamide concentrations. Yeast cells were treated with a series of concentrations of diamide (0, 1, 2, 3, 4 and 5 mM), respectively. Al^3+^ in each sample was quantified by ICP-OES after 2 h; (**b**) Al^3+^ accumulation over a time course under 5 mM diamide. Yeast cells were treated with 5 mM diamide and samples were then taken over a time course (0, 20, 40, 60, 80, 100 and 120 min). Non-treated yeast samples were used as controls. Al^3+^ in all samples was quantified by ICP-OES; (**c**) Al^3+^ accumulation in yeast cells cultured in Al^3+^-spiked media. Yeast cells treated with diamide (5 mM) were cultured in the media with and without 50 μM Al^3+^. The samples were taken over the time course and processed in the same way as the previous experiment. Error bars represent standard deviation of two biological replicate measurements.

Out of these ionomic data, it is the diamide-induced Al^3+^ accumulation, a 50-fold increase against the control, which is the most striking. A series of experiments was carried out in order to examine the relationship between diamide treatment and Al^3+^ accumulation. The data demonstrated that diamide-induced Al^3+^ accumulation was dose and time dependent (Figure 3). Firstly, the intracellular Al^3+^ content was elevated along the increase of diamide concentrations (Figure 3a). The 2 mM treatment showed a 62% increase and 5 mM diamide resulted in a 6.4-fold Al^3+^ accumulation. Secondly, the time course data (Figure 3b) revealed that cells exposed to diamide for between 20 and 120 min had gradually increased intracellular Al^3+^ uptake. At the start of diamide treatment, there was no difference between the control and treated cells, and the length of exposure time required by diamide to cause *S. cerevisiae* accumulating Al^3+^ is approximately 20 min. At this point, yeast sample showed a 2-fold increase and by 120 min, Al^3+^ accumulation was over 8.8-fold. The finding was further confirmed with the Al^3+^-spiked media (Figure 3c). Aluminium contents were elevated to 2.1 and 3.1 folds at 100 and 120 min were observed in Al^3+^-spiked media plus 5 mM diamide compared to Al^3+^-spiked media alone. These results confirmed the ionomic finding, namely Al^3+^ accumulation is linked to diamide treatment.

As previously described, diamide is a sulfhydryl-oxidising oxidant, which results in formation of unnatural disulfide bonds in living organisms upon exposure. The stress condition caused by diamide is thus specifically termed as disulfide stress [15]. The reaction mechanism by which diamide causes non-native disulfide formation within or between cellular proteins involves two steps, *i.e.*, the free thiol group in a protein is first oxidised by diamide to form sulfenylhydrazine, which then further reacts with another free thiol compound or protein to form a disulfide complex and a hydrazine derivative.

### 2.3. Further Validation of the Linkage Between Diamide-Induced Disulfide Stress and Al^3+^ Accumulation

**Figure 4:**
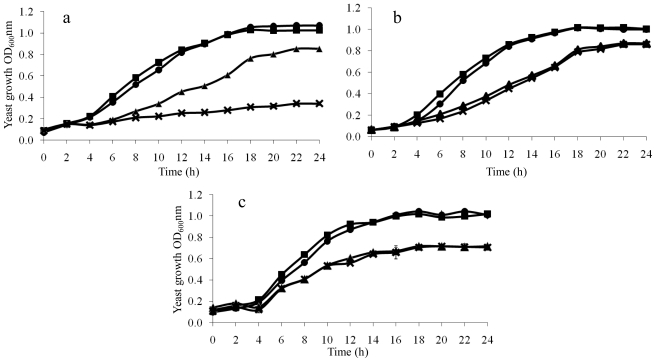
Relationships of diamide with Al^3+^, H_2_O_2_ with Al^3+^, and diamide with Cd^2+^. (**a**) Relationship of diamide with Al^3+^. Yeast cell growth was monitored in the media containing mild doses of diamide (0.4 mM) and Al^3+^ (0.8 mM) over a 24 h period (×). Diamide alone (●), Al^3+^ alone (▴) and the straight media (■) were used as controls; (**b**) Relationship of H_2_O_2_ with Al^3+^. The experiment was conducted as described previously. Treatment of the mild doses of H_2_O_2_ (1.0 mM) and Al^3+^ (0.8 mM) is denoted by (×), H_2_O_2_ (1.0 mM) alone by (●), Al^3+^ alone by (▴) and the straight media by (■); (**c**) Relationship of diamide with cadmium. Treatment of the mild doses of diamide (0.4 mM) combined with Cd^2+^ (25 μM) is denoted by (×), diamide (0.4 mM) alone by (●), Cd^2+^ alone by (▴) and the straight media by (■). Error bars represent standard deviation of two biological replicate measurements.

The specific relationship of diamide with Al^3+^ was further studied with yeast growth assays. A mild dose of diamide (0.4 mM), which did not inhibit yeast cell growth except for a minor adverse effect in the first 12 h, resulted in approximately 400% cell toxicity with little cell growth when it was combined with 0.8 mM Al^3+^, whereas 0.8 mM Al^3+^ alone caused a moderate 17% growth reduction at 24 h (Figure 4a). In contrast, a similarly mild dose of H_2_O_2_ (1 mM) led to little additional cell toxicity when combined with 0.8 mM Al^3+^, compared to Al^3+^ alone (Figure 4b). These results suggest that diamide-induced disulfide stress is specifically associated with Al^3+^ uptake. Diamide and Cd^2+^ also showed no synergistic effect (Figure 4c), which also argues that there is s strong causal link between diamide-induced stress and Al^3+^ uptake.

**Figure 5:**
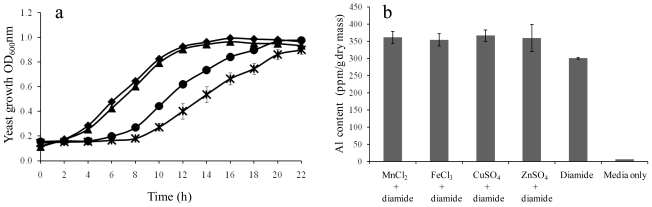
Potassium rescue against LAH and Al^3+^ accumulation of yeast cells in the presence of other metal ions. (**a**) Potassium rescue against LAH. Potassium rescue experiment was performed by the time course growth curves including yeast alone (■), yeast plus 5 g/L KCI (▴), yeast plus LAH (×), and yeast plus LAH and 5 g/L KCI (●). Error bars represent standard deviation of two biological replicate measurements; (**b**) Aluminium accumulation of yeast cells in the presence of other metal ions and diamide. Yeast cultures were treated with individual ion-containing compound as well as 5 mM diamide. These compounds were manganese dioxide, manganese chloride, ferric chloride, copper sulphate and zinc sulphate. A control culture without any element was treated with 5 mM diamide. A media control with neither element nor diamide was also included. Cultures were terminated after 2 h treatment and Al^3+^ contents were measured using ICP-OES.

In a transcriptomics study by Leichert, *et al.* [15], the responses to diamide in *Bacillus subtilise* were found to be a complex combination of different regulatory networks related to oxidative stress, heat stress, heavy metal detoxification and growth inhibition. Such an up-regulation of metal detoxification pathways under diamide stress could well be due to the increased uptake of metal ions like Al^3+^, as revealed in this study in yeast cells, although the abovementioned study did not measure any levels of metal ions. The reason for diamide-induced Al^3+^ influx could be due to the oxidation of free thiol groups in transmembrane proteins by diamide, which in turn caused membrane damage and hence Al^3+^ influx. Another possibility is that Al^3+^ was taken up via the transporters for iron, zinc, copper and manganese. To investigate this hypothesis, yeast was first incubated with an excess of zinc, iron, copper and manganese, respectively, and then treated with diamide to see if these metal ions could block Al^3+^ influx. The results demonstrated that there was little difference between the treatment of element plus diamide and diamide alone (Figure 5b), indicating transporters for these elements were not used in Al^3+^ uptake. The data also imply there might be unique Al^3+^ transporters in yeast cells. Until now, however, no such channel has been reported in yeast, plant or animal. Little is known regarding the specifics of aluminium adsorption and its metabolism. In addition, apart from the finding that iron and the other metal ions did not block the Al^3+^ entry, they actually caused a minor increase of Al^3+^ accumulation instead (Figure 5a). This agrees with the published observation that Al^3+^ and iron uptake were enhanced by one another in human glial cell lines through a carrier-mediated mechanism [18]. Such a concurrent uptake of Al^3+^ and iron could augment the abnormal cellular metabolism and hence cause disease [19].

These findings strongly establish that diamide-induced stress indeed triggers Al^3+^ accumulation. This may shed light on the pathogenesis of human neurodegenerative diseases. Aluminium mineral is highly abundant in our environment, constituting approximately 7% of the earth’s crust [20]. Its apparent non-toxicity due to its insolubility at neutral pH can be changed as the acidity of soil becomes prevalent. The increased solubility of aluminium leads to an increased abundance of Al^3+^ ion which is potentially harmful to plants, animals and human being [4,21]. As is known, Al^3+^ is omnipresent in foods, commercial beverages and water. High quantities of aluminium are also present in medications such antacids, buffered aspirin and adjuvant. Al^3+^ can bind to both inorganic phosphate ions and various organic phosphate compounds. Because the cell membrane and human central nervous system within the brain are rich in phospholipid, Al^3+^ might be involved in neurodegenerative diseases such as Alzheimers disease. Indeed, experimental data has demonstrated that Al^3+^ is present in neural degenerative body—the amyloid plaque [22–24]. Any patients compromised with disulfide stress would be more vulnerable to Al^3+^ accumulation and its potential toxic effects such as cytotoxicity and protein aggregation.

### 2.4. Growth Rescue of LAH-Treated Yeast Cells by Additional K^+^

The depletion of potassium upon LAH exposure was also further validated by a rescue experiment to explore if additional K^+^ could compensate LAH-induced K^+^ depletion and in turn ameliorate LAH-induced stress. Results (Figure 5a) demonstrated that additional K^+^ indeed increased yeast cell growth by approximately 26% under LAH treatment in comparison to LAH alone, while additional magnesium or sodium had no beneficial effect on cell growth under LAH treatment (data not shown). Hence, the result confirmed the relationship between LAH and K^+^ revealed by the initial ionomic profiling. The implications of K^+^ involvement in such antioxidant activity are two-fold. Firstly, K^+^ should be considered as a component in antioxidant development; secondly, the underlining molecular mechanism for its antioxidant activity should be investigated in future studies, through transcriptomics for example, so that a full picture of ion, gene and protein interactions could be uncovered.

## 3. Experimental Section

### 3.1. Yeast Strain and Culture Conditions

The *S. cerevisiae* strain BY4743 (MATa/α his3Δ1/his3Δ1 leu2Δ0/leu2Δ0 met15Δ0/MET15 lys2Δ0/LYS2 ura3Δ0/ura3Δ0) used in this study was obtained from EUROSCARF (Frankfurt, Germany) [25].

BY4743 was streaked from a frozen glycerol stock to the YEPD agar plate containing 1% yeast extract, 2% peptone, 2% d-glucose and 2% agar, and grown at 30 °C for 48 h. Yeast culture was initiated by inoculation of a single colony from the agar plate into the minimal medium containing 2% d-glucose, 0.17% yeast nitrogen base with neither ammonium sulphate nor amino acids, 0.5% ammonium sulphate, and supplemented with 10 mg L^−1^ adenine, 50 mg L^−1^ l-arginine, 80 mg L^−1^ l-aspartic acid, 20 mg L^−1^ l-histidine HCl, 50 mg L^−1^ l-isoleucine, 100 mg L^−1^ l-leucine, 50 mg L^−1^ l-lysine HCl, 20 mg L^−1^ l-methionine, 50 mg L^−1^ l-phenylalanine, 100 mg L^−1^ l-threonine, 50 mg L^−1^ l-tryptophan, 50 mg L^−1^ l-tyrosine, 140 mg L^−1^ l-valine and 20 mg L^−1^ uracil. The culture was shaken at 150 rpm at 30 °C. Yeast growth was monitored by measuring the optical density at 600 nm (OD_600_) using a spectrophotometer. Yeast in peroxynitrite experiments was cultured in phosphate-buffered minimal medium lacking tyrosine, and methionine.

### 3.2. Oxidants

Hydrogen peroxide, cumene hydroperoxide, diamide and menadione were purchased from Sigma-Aldrich (USA). Linoleic acid hydroperoxide (LAH) and peroxynitrite were synthesised as follows. The synthesis of LAH was carried out as described previously by incubating linoleic acid (1.0 mM) with soybean lipoxygenase (5000 U) in 0.1 M tetra-sodium borate buffer (pH 9.0) at room temperature with vigorous stirring for 30 min [26]. The enzyme catalyzes abstraction of the hydrogen on the intervening methylene group between the double bonds in linoleic acid, leading to the formation of LAH. LAH was purified by loading the reaction mixture onto an end-capped C18 reverse-phase Sepak cartridge (Waters, Australia), followed with a methanol elution step. The concentration and purity of LAH were determined spectrophotometrically (λ = 234 nm; 1 OD_234_ = 25,000 M^−1^ cm^−1^). The synthesized LAH in the study was 14 mM in concentration.

Peroxynitrite was synthesized using isoamyl nitrite and hydrogen peroxide. Sodium hydroxide (2 M) in 38.5 mL volume was mixed with 11.5 mL 30% H_2_O_2_ (Sigma-Aldrich, USA) and 0.5 mL 0.5 M EDTA in a 500 mL bottle. Isoamyl nitrite (25 mL) was then added to the solution while stirring at 4 °C. The bottle was sealed with parafilm and stirred overnight. The synthesis was manifested by changing colour from yellow to orange. The excess of isoamyl nitrite was separated in a funnel, and the aqueous orange layer containing peroxynitrite was collected and further purified by mixing with an equal volume of chloroform. Manganese oxide (0.25 g) was added to the peroxynitrite fraction for scavenging residual H_2_O_2_. Peroxynitrite was finally obtained by filtration to remove manganese oxide. The concentration and purity was determined by reading absorbance at 302 nm (1 OD_302_ = 1670 M^−1^ cm^−1^). The synthesized peroxynitrite concentration in the study was 1000 mM.

### 3.3. Determination of Arresting Concentrations of the Oxidants

The oxidant treatment was performed by addition of oxidant during the exponential growth phase (OD_600_ = 1.0) of yeast strain BY4743, based on the BY4743 growth curve. The arresting concentration for each oxidant was determined by assaying a series of the oxidant concentrations with yeast of OD_600_ 1.0. Oxidant at varying concentrations was added to each flask containing 20 mL yeast culture. These cultures were subsequently incubated at 30 °C with shaking (150 rpm). Aliquots (100 μL) were removed at 20 min intervals over 2 h, and after dilution (1:4000) spread onto YEPD agar plates in duplicate. The YEPD plates were incubated at 30 °C, for a minimum of 36 h before the number of colonies present on each plate was counted. The colony numbers for each concentration of the oxidant was graphed and the arresting concentration for each oxidant was determined.

### 3.4. Oxidant Treatment and ICP-AES Analysis

Seven flasks of BY4743 yeast, in 100 mL volume of minimal medium, were grown to an OD_600_ of 1.0. Six were treated with each of the oxidants respectively at the determined arresting concentrations, while one flask was used as the control with no oxidant added. Each culture was subsequently split into two 50 mL tubes at the end of 2 h treatment and processed for ICP-OES analysis. The yeast sample washing procedure was modified from the method of Eide *et al.* [8]. The samples were firstly centrifuged at 4000 g for 10 min at 15 °C. Cell pellets were then washed twice with 30 mL of 2 μM ethylenediaminetetraacetic acid (EDTA) disodium solution, pH 8.0, and followed with two more washes with 30 mL deionized H_2_O. Cell pellets were finally resuspended in 1 mL water and transferred into pre-weighed Eppendorf tubes. The samples were spun down at 10,000 g for 5 min, and cell pellets were dried at 70 °C for 4 h. The dry mass for each sample was determined gravimetrically. Medium supernatant samples were also taken for ICP analysis.

Yeast samples were digested prior to analysis by ICP-OES (Varian 720-ES series). Each sample was digested in 1 mL 65% nitric acid (Suprapur Merck) for 24 h at 25 °C. The samples were then vortexed and centrifuged for 10 min at 10,000 g. Each sample was diluted 1:100 (100 μL sample in 9.9 mL MilliQ water). The Varian 720-ES consisted of an axial torch configuration and sample introduction system equipped with a Seaspray nebuliser and Twister cyclonic baffled spray chamber. The sample introduction system was fitted with a Y-piece which incorporated on-line dilution of samples and standards with yttrium internal standard solution. Data analysis was completed with ICP Expert II software. ICP standard element solutions for Zn^2+^, Fe^2+/3+^, Al^3+^, Cu^+/2+^, Mn*^n^*^+^ at 0, 0.05, 0.2, 0.5, 1.0 ppm were prepared via appropriate dilutions of standard solutions in ultrapure water and nitric acid at the final concentration of 6.5% (v/v). Also prepared were Na^+^, Mg^2+^ and Ca^2+^ at 0, 10, 25, 50 and 100 ppm as well as K^+^ at 0, 50, 100, 200 and 300 ppm. Media samples were also analysed without nitric acid digestion at 1:5 dilution in water.

### 3.5. Characterization of Aluminium Accumulation over a Range of Diamide Concentrations and over a Time Course

BY4743 (6 × 100 mL cultures) were grown to an OD_600_ of 1.0. Each flask was then split into two lots, one serving as the control and the other received diamide of 0, 1, 2, 3, 4 and 5 mM respectively. Two replicates with 25 mL each were taken from each flask prior to addition of diamide. The cultures were then treated and incubated for 1 h. Duplicate samples of 25 mL from both the treated and the control were subsequently analysed by ICP-OES.

A time course study of diamide effect on cellular Al^3+^ content was set up as follows. A single yeast BY4743 colony was inoculated into 50 mL minimal media, allowed to grow overnight at 30 °C, 150 rpm. The pre-culture was diluted to an OD_600_ 0.4 in 1400 mL media and then divided into 4 aliquot flasks, which consisted of two control flasks and two 5 mM diamide flasks both of 350 mL. The cultures were incubated at 30 °C with shaking until OD_600_ reached 1.0. Aliquots of 25 mL replicates were removed at regular intervals of 0, 20, 40, 60, 80, 100 and 120 min. A total of 56 samples was collected which represented 14 samples for each treatment. This study was also carried out using the Al^3+^ (50 mM)-spiked media plus and minus 5 mM diamide.

### 3.6. Relationships of Diamide with Al^3+^, H_2_O_2_ with Al^3+^, and Diamide with Cd^2+^

The relationship of diamide and Al^3+^ was further investigated using mild doses of diamide (0.8 M) and Al^3+^ (0.8 mM) in 96-well plate. The plates were incubated in 30 °C incubator with shaking at 750 rpm. Yeast growth was monitored by reading OD_600_ from the starting point and at 2 h intervals to the end of 24 h period using a microplate reader (Multiskan EX, Thermo Electron, USA). The relationships of diamide (0.8 mM) *versus* Cd^2+^ (25 μM) and H_2_O_2_ (1 mM) *versus* Al^3+^ (0.8 mM) were characterised in parallel in order to delineate if diamide and Al^3+^ relationship is specific.

### 3.7. Al^3+^ Uptake of Yeast Cells in the Presence of Other Elements

Diamide-induced Al^3+^ uptake was measured after growth in the presence of other cations-manganese, iron, copper and zinc. Yeast culture for each treatment was carried out in 100 mL volume. Each culture was allowed to grow to OD_600_ of 1.0 and then 10 mg of each individual ion-containing compound (manganese chloride, ferric chloride, copper sulphate and zinc sulphate) as well as 5 mM diamide was added. A control culture without any element was treated with 5 mM diamide only. A media control with neither element nor diamide was also included in the study. Cultures were terminated after 2 h treatment. Each culture was split into two 50 mL replicates and centrifuged. The yeast pellets were washed, dried, weighed and analysed with ICP-OES.

## 4. Conclusions

In this study, oxidants were clearly shown to affect levels of cellular metal ions in yeast cells. Pronounced and distinct changes were observed against varying oxidative stress conditions. Such oxidant-specific responses clearly demonstrate the uniqueness of particular oxidant-cell interactions. The finding that diamide-induced stress caused a marked elevation of Al^3+^ accumulation establishes for the first time the linkage between disulfide stress and Al^3+^ accumulation. Further investigation is needed in order to delineate the molecular mechanism responsible for the diamide-induced Al^3+^ uptake and cell toxicity. The delineation of potassium’s role in protecting cells from LAH-induced stress is significant to basic antioxidant research and industrial applications such as yeast storage and fermentation. In essence, this study has revealed a number of exciting cues on the specific oxidant-metal ion interactions for future investigation, particularly in the contexts of relevant medical and biological problems.
